# Predicting complicated appendicitis based on clinical findings: the role of Alvarado and Appendicitis Inflammatory Response scores

**DOI:** 10.1007/s00423-022-02533-5

**Published:** 2022-05-11

**Authors:** F. Haak, O. Kollmar, A. Ioannidis, J. E. Slotta, M. B. Ghadimi, T. Glass, M. von Strauss und Torney

**Affiliations:** 1 Department of Visceral Surgery, University Centre for Gastrointestinal and Liver Diseases, St. Clara Hospital and University Hospital Basel, Spitalstrasse 21, 4031 Clarunis Basel, Switzerland; 2 Clinical Biostatistics and Data Management Group, Swiss Tropical and Public Health Institute Basel, Basel, Switzerland; 3 General, Visceral and Transplantation Surgery, Westpfalz Klinikum, Kaiserslautern, Germany; 4 Department of General, Visceral and Pediatric Surgery, University Medical Center, Goettingen, Goettingen, Germany

**Keywords:** Appendicitis, Complicated appendicitis, Uncomplicated appendicitis, Appendectomy, Clinical scoring systems, Imaging

## Abstract

**Purpose:**

The pre-interventional differentiation between complicated and uncomplicated appendicitis is decisive for treatment. In the context of conservative therapy, the definitive diagnosis of uncomplicated appendicitis is mandatory. This study investigates the ability of clinical scoring systems and imaging to differentiate between the two entities.

**Methods:**

This is a retrospective analysis of two cohorts from two tertiary referral centers in Switzerland and Germany. All consecutive patients underwent appendectomy between January 2008 and April 2013 (in the first cohort) or between January 2017 and June 2019 (the second cohort). Exclusion criteria did not apply as all patients found by the database search and received an appendectomy were included. Diagnostic testing and calculation of a receiver operating curve were performed to identify a cutoff for clinical scores that resulted in a minimum sensitivity of 90% to detect complicated appendicitis. The cutoff was combined with additional diagnostic imaging criteria to see if diagnostic properties could be improved.

**Results:**

Nine hundred fifty-six patients were included in the analysis. Two hundred twenty patients (23%) had complicated appendicitis, and 736 patients (77%) had uncomplicated appendicitis or no inflammation. The complicated appendicitis cohort had a mean Alvarado score of 7.03 and a mean AIR of 5.21. This compared to a mean Alvarado of 6.53 and a mean AIR of 4.07 for the uncomplicated appendicitis cohort. The highest Alvarado score with a sensitivity of > 90% to detect complicated appendicitis was ≧ 5 (sensitivity = 95%, specificity 8.99%). The highest AIR score with a sensitivity of > 90% to detect complicated appendicitis was ≧ 3 (sensitivity 91.82%, specificity 18.53). The analysis showed that additional CT information did not improve the sensitivity of the proposed cut-offs.

**Conclusion:**

AIR and Alvarado scores showed limited capability to distinguish between complicated and uncomplicated appendicitis even with additional imaging in this retrospective cohort. As conservative management of appendicitis needs to exclude patients with complicated disease reliably, appendectomy seems until now to remain the safest option to prevent undertreatment of this mostly benign disease.

## Introduction

Appendectomy has been the gold standard in suspected appendicitis for more than a century regardless of the severity of the disease or the particular patient condition. It was uniformly thought to be a progressive disease that inevitably led to perforation and sepsis if not resected promptly. Consequently, diagnostic laparoscopies and appendectomies were performed for uninflamed specimens in fear of non-recognition of subtle disease. At the same time, surgery is a safe and effective treatment. Finally, surgery is a definitive therapy preventing the recurrence of the disease. Nevertheless, several randomized controlled trials and cohort studies have recently demonstrated the effectiveness of antibiotic treatment for uncomplicated appendicitis [1–7]. There is also evidence that the two entities (uncomplicated vs. complicated appendicitis) are different pathophysiological diseases. [8] This has led to the question of how surgeons and emergency physicians can reliably distinguish between complicated and uncomplicated diseases by clinical means and imaging. There is no universal definition that clearly defines an inflammation of the appendix as complicated or uncomplicated. While some authors solely define a perforation as complicated, others propose a more elaborate definition. [9] For example, the European Association of Endoscopic Surgery (EAES) defines uncomplicated appendicitis as an inflammation of the appendix without signs of gangrene, perforation, intra-peritoneal purulent fluid, contained phlegmon or intra-abdominal abscess, while any of the features mentioned above would be classified as complicated appendicitis. [10]

While there is a necessity for source control in the case of complicated appendicitis with surgery by removing the appendix, the paradigm of resection for uncomplicated appendicitis has been challenged with the introduction of definitive antibiotic therapy. [10, 11] This is also reflected in the recommendations of national and international guidelines. [12, 13]

A diagnostic dilemma for complicated appendicitis remains with the clear indication for surgery. The definition can only be made in retrospect once the cut has been made and clear visualization of the situation has been established with the potential for unnecessary surgery. Adjuncts exist like clinical scores or radiologic imaging to help the clinician decide a priori if surgery is warranted or not. [14]

The Alvarado score was developed in 1986 and consists of 6 clinical items and two laboratory markers, allowing a maximum allocation of 10 points to a patient with suspected appendicitis. [15] Similarly, the Appendicitis Inflammatory Response (AIR) score was developed in 2008 and consists of 4 clinical items and three laboratory markers allocating a maximum of 12 points. [16] The scoring systems help interpret the clinical situation, with higher scoring being associated with a higher likelihood of acute appendicitis but not distinguishing between complicated and uncomplicated disease.

Therefore, our study aims to determine whether the two clinical scoring systems can help predict complicated appendicitis. Additionally, we investigate the added value of imaging after using clinical scoring systems.

## Methods

This retrospective analysis evaluated two cohorts from two tertiary referral centers in Switzerland and Germany: All consecutive patients who underwent appendectomy between January 2008 and April 2013 (in the first cohort) or between January 2017 and June 2019 (the second cohort). Exclusion criteria did not apply as all patients found by the database search and received an appendectomy were included. Comorbidities were not examined. Data were obtained by chart review, and results were checked systematically by independent members of the research team. Appendicitis was defined by the recognition of inflammation on the histopathology report. Complicated appendicitis was defined using a prerequisite (appendicitis confirmed by histopathology) and additional information (abscess found intraoperatively, perforation found intraoperatively, generalized or localized peritonitis found intraoperatively, or perforation confirmed by histopathology) to match the EAES definition for complicated appendicitis as best as possible. [10] If one of the abovementioned criteria was found, appendicitis was defined as complicated. If no additional criteria were found, the appendicitis was considered uncomplicated. Appendicitis was defined as complicated in a CT scan if an abscess or perforation was identified. If none of these two criteria wass met, appendicitis was defined as uncomplicated in the CT scan. Board-certified radiologists with at least 5 years of experience validated radiological examinations.

Data were summarized using medians and interquartile ranges for continuous variables and frequencies and percentages for categorical variables. Associations between two categorical variables were done using a chi-square test or Fisher’s exact test. Associations between the two scores were done using Pearson’s correlation coefficient.

The association between the median Alvarado and AIR scores and diagnosis of appendicitis (without differentiation between complicated and uncomplicated) intraoperatively and by histopathology was compared using a Wilcoxon Rank-Sum test.

Alvarado and AIR scores were calculated according to the original publication. [15, 16] There were missing data for Alvarado and AIR calculations in both cohorts to a certain degree. (Please see additional references Table 4 for the exact amount of missing variables.) A “Alvarado/AIR best” was calculated to account for missing data by replacing every missing diagnostic item with the least number of possible points, thereby limiting false patient exclusion with complicated appendicitis due to missing values and underrepresentation of disease severity by value imputation. Additionally, an “Alvarado/AIR worst” was calculated to show the potential frame where the true values must lie (see additional references Table 5 for all values).

We performed diagnostic testing and calculated a receiver operating curve to identify the cutoff for the scores that resulted in a minimum sensitivity of 90% to detect complicated appendicitis. We then combined this cutoff with additional diagnostic criteria like the results of a CT scan and sonography to see if diagnostic properties could be improved. Furthermore, diagnostic testing was repeated stratified by gender, BMI, and time from diagnosis to surgery. Sensitivity, specificity, likelihood ratios, area under the curve, and resulting 95% confidence intervals were reported.

All analyses were done using Stata version 15 (StataCorp, Texas, USA).

## Results

Nine hundred fifty-six patients who received an appendectomy were included in the analysis. Two hundred twenty patients (23%) had complicated appendicitis, and 736 patients (77%) had uncomplicated appendicitis or no inflammation. Three hundred six appendectomies were performed in the Swiss and 650 in the German centre.

In the complicated appendicitis cohort, 91 patients (41%) were female and 129 male (59%). The uncomplicated appendicitis cohort had more females (384 or 52%) than males (48%). The patients in the uncomplicated appendicitis cohort were younger (median age 26 vs. 41). The patients’ weight between the cohorts was comparable (median BMI 24.9 vs. 23.3) (Table 1).

**Table 1:** Basic demographics

	Complicated appendicitis	Uncomplicated appendicitis + no appendicitis	Total
N	220 (100%)	736 (100%)	956 (100%)
Gender Female Male	91 (41%) 129 (59%)	384 (52%) 357 (48%)	475 (49%) 486 (51%)
Age Median (IQR)	41 (22–62.5)	26 (18–38)	28 (19–43)
BMI Median (IQR)	24.9 (21.2–27.9)	23.3 (20.6–27.1)	23.5 (20.7–27.2)

Sonography was performed in most patients in both cohorts (81% of the complicated appendicitis patients received sonography and 90% of the uncomplicated/no appendicitis patients received sonography). Sixty percent of the performed sonographies for the complicated appendicitis group were diagnostic for acute appendicitis.

A CT scan was performed in 29% of the complicated appendicitis cases compared to 9% of the uncomplicated/no appendicitis cases. Of the 64 CT scans performed in the complicated group, 98% of the scans were diagnostic for acute appendicitis. Of the 68 CT scans performed in the uncomplicated/no appendicitis group, 97% of the scans showed acute appendicitis. Twenty-two of the 64 CT scans (35%) performed in the complicated appendicitis group specified appendicitis as being complicated (additional references Table 2).

**Table 2:** Intraoperative findings

	Complicated appendicitis	Uncomplicated appendicitis + no appendicitis	Total
*N*	220 (100%)	736 (100%)	956 (100%)
Appendicitis			
Yes	218 (218/220 = 99%)	649 (649/736 = 89%)	867 (867/956 = 90%)
No	0 (0/220 = 0%)	80 (80/736 = 11%)	80 (80/956 = 8%)
Missing Info	2 (2/220 = 1%)	7 (7/736 = 1%)	9 (9/956 = 1%)
Abscess			
Yes	17 (17/220 = 8%)	0 (0/736 = 0%)	17 (17/956 = 2%)
No	203 (203/220 = 92%)	736 (736/736 = 100%)	939 (939/956 = 98%)
Perforated			
Yes	154 (154/220 = 68%)	5 (5/736 = 1%)*	159 (159/956 = 17%)
No	66 (66/220 = 30%)	731 (731/736 = 99%)	797 (797/956 = 83%)
Peritonitis – generalized			
Yes	12 (12/220 = 5%)	0 (0/736 = 0%)	12 (12/956 = 1%)
No	208 (208/220 = 95%)	736 (736/736 = 100%)	944 (944/956 = 99%)
Peritonitis – localized			
Yes	128 (128/220 = 58%)	8 (8/736 = 1%)*	136 (136/956 = 14%)
No	92 (92/220 = 42%)	728 (728/736 = 99%)	820 (820/956 = 86%)

^*^Cases were classified as uncomplicated or no appendicitis as histopathology did not confirm the diagnosis of appendicitis and source of intraoperative suspected perforation and peritonitis was found to be other than an appendix

Intraoperatively, 80 patients (which corresponds to 11% of the uncomplicated/no appendicitis cohort or 8% overall) were described as not being inflamed/no appendicitis (Table 2). Preoperatively 75 of these patients received sonography. Fifty (66.7%) of these sonographies did not indicate appendicitis. Eighteen (24%) indicated appendicitis. Only 1 CT scan was performed for this subgroup which showed appendicitis. 61.4% of the complicated appendicitis cases were completed laparoscopically, 75.2% of the uncomplicated appendicitis cases, and 72.8% of the no appendicitis cases.

Histopathologically, 191 cases (26%) of the uncomplicated/no appendicitis group were shown not to have any inflammation (Table 3).

**Table 3:** Histopathological findings

	Complicated appendicitis	Uncomplicated appendicitis + no appendicitis	Total
*N*	220 (100%)	736 (100%)	956 (100%)
Appendicitis			
Yes	220 (220/220 = 100%)	545 (545/736 = 74%)	765 (765/956 = 80%)
No	0 (0/224 = 0%)	191 (191/736 = 26%)	191 (191/956 = 20%)
Perforated			
Yes	119 (119/220 = 54%)	0 (0/736 = 0%)	119 (119/956 = 12%)
No	101 (101/220 = 46%)	736 (736 /736 = 100%)	837 (837/956 = 88%)

There was no difference between the duration of time from arrival at the emergency department to the operation between the complicated and uncomplicated/no appendicitis groups (complicated appendicitis group median = 7.4 h and uncomplicated/no appendicitis median = 8). The same applied for the time of OR registration and operation (complicated appendicitis group median = 3.4 h vs. 4 h for the uncomplicated/no appendicitis group).

Further details on the postoperative course of treatment are displayed in additional references Table 1.

### Clinical scores

Concerning the association of calculated scores with appendicitis, we could confirm that higher scores correlate with the diagnosis of appendicitis compared to lower scores predicting the intra and postoperative finding of an uninflamed appendix. The *Z*-value for Alvarado was − 4.692 (*p* < 0.001) and − 4.994 for AIR (*p* < 0.001).

We did not find a significant association between Alvarado scores and whether a CT was performed (*p* = 0.75), but there was a significant association between the AIR score and whether a CT was performed (*p* < 0.001).

### Primary outcome

The complicated appendicitis cohort had a mean Alvarado score of 7.03 (SD 1.40). This compared to a mean Alvarado of 6.53 (SD 1.39) for the uncomplicated appendicitis group and a mean Alvarado of 5.58 (SD 1.39) for the no appendicitis group. The mean AIR for the complicated appendicitis cohort was 5.21 (SD 2.0). The mean AIR for the uncomplicated appendicitis group was 4.07 (SD 1.77) and 2.96 (SD 1.70) for the no appendicitis group.

**Fig. 1:**
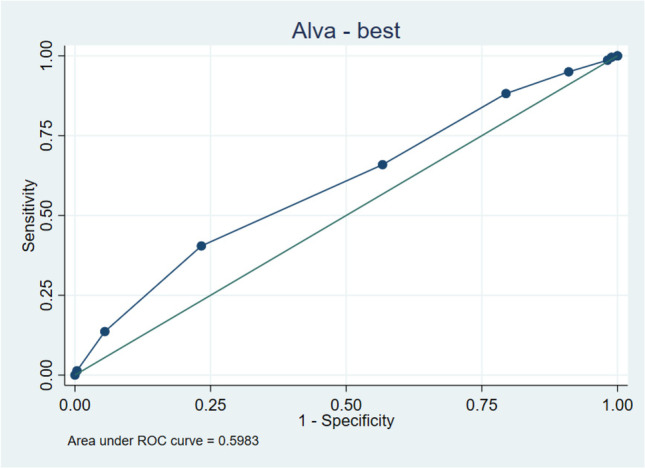
ROC (receiver operating curve) for Alvarado

**Fig. 2:**
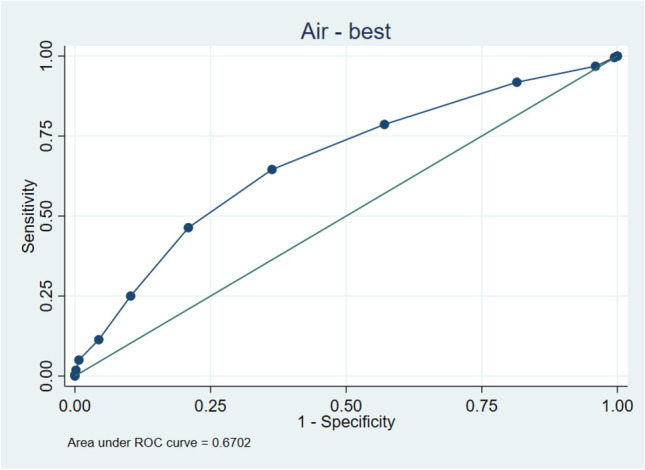
ROC (receiver operating curve) for AIR (appendicitis inflammatory response)

The highest Alvarado score with a sensitivity of > 90% to detect complicated appendicitis was ≧ 5 (sensitivity = 95%, specificity 8.99%, LR + 1.044, LR − 0.556). The highest AIR Score with a sensitivity of > 90% to detect complicated appendicitis was ≧ 3 (sensitivity 91.82%, specificity 18.53%, LR + 1.127, LR − 0.442) (please refer to Table 4 for complete cutoff calculation results and Figs. 1 and 2 for ROC curves.

**Table 4:** Cutoff calculation via receiver operating curve (ROC)

Cut point	Sensitivity	Specificity	LR +	LR −
Alvarado: cutoff calculation				
≥ 2	100.00%	0.00%	1.000	
≥ 3	99.55%	1.10%	1.007	0.413
≥ 4	98.64%	1.83%	1.005	0.743
≥ 5	95.00%	8.99%	1.044	0.556
≥ 6	88.18%	20.55%	1.110	0.575
≥ 7	65.91%	43.30%	1.163	0.787
≥ 8	40.45%	76.70%	1.736	0.776
≥ 9	13.64%	94.50%	2.477	0.914
≥ 10	1.36%	99.63%	3.716	0.990
> 10	0.00%	100.00%		1.000
ROC computation statistics				
Obs = 765, area = 0.598, Std. Err. 0.0225, 95% Conf. interval = 0.554–0.642				
AIR: cutoff calculation				
≥ 0	100.00%	0.00%	1.000	
≥ 1	99.55%	0.55%	1.001	0.826
≥ 2	96.82%	4.04%	1.009	0.788
≥ 3	91.82%	18.53%	1.127	0.442
≥ 4	78.64%	42.94%	1.378	0.498
≥ 5	64.55%	63.67%	1.777	0.557
≥ 6	46.36%	79.08%	2.217	0.678
≥ 7	25.00%	89.72%	2.433	0.836
≥ 8	11.36%	95.60%	2.581	0.927
≥ 9	5.00%	99.27%	6.813	0.957
≥ 10	1.82%	99.82%	9.909	0.984
≥ 11	0.45%	100.00%		0.995
> 11	0.00%	100.00%		1.000
ROC computation statistics				
Obs = 765, area = 0.670, Std. Err. 0.022, 95% Conf. interval = 0.628–0.713

If the Alvarado scores were stratified by gender, the diagnostic properties are slightly better in women (AUC = 0.62 versus 0.59) leading to a cutoff ≥ 6 to achieve a sensitivity > 90% in female patients. Similarly, the AIR scores had slightly better diagnostic properties in women than in men, but here the cutoff did not change to achieve a sensitivity of > 90%.

### Secondary outcome

The analysis showed that additional information from a CT scan only marginally improved or did not improve the sensitivity of the proposed cutoff. In the investigated cohorts, the CT scan added + 0.5% sensitivity to the Alvarado cutoff of ≧ 5 and + 0% sensitivity to AIR cutoff of ≧ 3 (Table 5).

**Table 5:** Improvement of diagnostic properties of scores by imaging

Test	Sensitivity %	Specificity %
Alvarado		
Alvarado ≥ 5	95.0	9.0
Alvarado ≥ 5 + CT	95.5	8.8
Alva ≥ 5 + sonography	95	9.0
AIR		
AIR ≥ 3	91.8	18.5
AIR ≥ 3 + CT	91.8	18.5
AIR ≥ 3 + sonography	92.3	18.5

Finally, looking at the association between time from presentation to surgery based on four categories (operation within 2 h, 2–6 h, 6–24 h, > 24 h) and the diagnosis of complicated appendicitis, there was no evidence of a correlation of delayed surgery and the development of complicated disease (chi-square 5.246; *p*-value of 0.155; additional references Table 3).

## Discussion

The present analysis does not support the routine use of established clinical scoring systems for appendicitis to distinguish between complicated and uncomplicated appendicitis. The cutoffs with acceptable sensitivity have a very low specificity, which in the end would not lead to a useful identification of eligible patients because the calculated cutoffs lie within the intermediate (Alvarado) or low (AIR) risk categories for appendicitis defined by the original publications for the clinical scores. [15, 16] Recommended management for the low-risk category is outpatient follow-up. Data exists showing the safety and effectiveness of this strategy. [17] Proposing a cutoff AIR of 3 in light of this strategy is not plausible.

Our results fall in line with the work of Dieters et al., who solely investigated the role of Alvarado for distinguishing between complicated and uncomplicated appendicitis in a retrospective study in 2019. [18] Their cohort was significantly smaller and depicts a different population with a higher proportion of complicated appendicitis than our cohort and what has been reported in the literature. [19] Additionally, Lietzén et al. showed that clinical findings (temperature) and laboratory markers (C-reactive protein and white blood cell count) cannot differentiate between complicated and uncomplicated appendicitis. [20] These factors are part of the scores we evaluated but only represent a small portion of the involved components.

Published results demonstrate that clinical scores should be used as part of a step-up approach to guide the further diagnostic and therapeutic pathway. [21] It currently has no role as a discriminator between uncomplicated and complicated appendicitis or between non-operative treatment and upfront appendectomy.

Interestingly, contrary to the previously published series, our data shows that CT scan or sonography does not improve the sensitivity of the established scoring systems in detecting complicated appendicitis. [22] This contradicts the recent OPTICAP study which showed that a low-dose CT scan could distinguish reliably between complicated and uncomplicated appendicitis. [23] Of note, the OPTICAP study entailed only a cohort of 60 patients.

In the context of this debate, we would like to reiterate that diagnostic laparoscopy can be used as a reliable and safe method to differentiate between complicated and uncomplicated appendicitis without the short comings of radiation exposure with computer tomography or limited availability with magnetic resonance imaging.

Contrary to other studies, our analysis could not show a higher rate of complicated appendicitis with longer delay to surgery, questioning whether every case of appendicitis can be genuinely considered a surgical emergency. [24, 25] This especially has an implication for reduced operation capacity in crises as the COVID pandemic. [26]

Several RCTs have shown that conservative antibiotic therapy seems to be a valid option for uncomplicated appendicitis. [1, 2, 27] Most recently, the prospective CODA trial from the USA reported the use of antibiotic therapy in a large cohort of all comers in emergency departments without particularly challenging inclusion criteria and limiting treatment to uncomplicated disease. [7] In this study, in the subgroup of patients with pretreatment evidence of complicated disease (on CT), failure rates of the conservative arm reached nearly 50% showing that it remains paramount to reliably exclude complicated appendicitis before the start of any conservative treatment. The commentary from Jacobs also states that surgery remains the standard of therapy for appendicitis. [28] A similar fact is drawn from the Prospero trial showing a treatment failure of the conservative group of 26.5% within 1 year. [27]

### Limitations

The communicated results must be seen in the context of the retrospective nature of this study. Due to this, we have a preselected cohort of appendicitis patients. A prospective study would include patients with unclear abdominal symptoms and demonstrate a more realistic population of patients. But because the question at hand is the differentiation between complicated and uncomplicated appendicitis, our predefined cohort is sufficient for the analysis. Additionally, we have to mention the amount of missing data. This is due to insufficient documentation of emergency files and the individual habits of physicians. On top of there is no standardized evaluation of patients with lower abdominal pain at our emergency department, adding to this problem. For this study, we merged two databases from two different centers. The resulting incongruity of data was minimal due to the matching query criteria.

## Conclusion

Our data shows that—at least the established—clinical scoring systems seem to be unable to distinguish between complicated and uncomplicated appendicitis reliably. Future studies will need to identify safe ways to exclude complicated disease.
